# All the Risk You Cannot See: Residual Risk of Airborne PM_2.5_ After CABG∗

**DOI:** 10.1016/j.jacadv.2023.100782

**Published:** 2023-12-20

**Authors:** Graham H. Bevan

**Affiliations:** Division of Cardiology, University of Washington, Seattle, Washington, USA

**Keywords:** ambient air pollution, coronary artery disease

**Figure:**
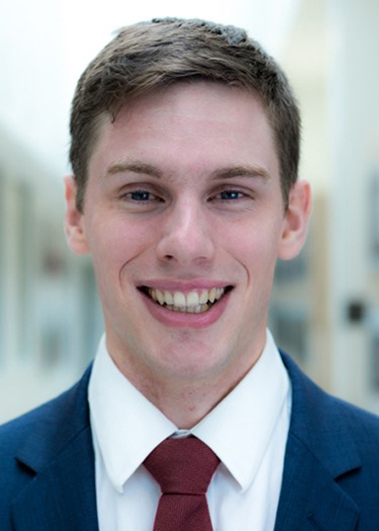

Particulate matter (PM) air pollution <2.5 μm in diameter (PM_2.5_) has well-established adverse effects on cardiovascular health.^1^ By and large, exposure to this risk factor has steadily declined in North America from an estimated population-weighted annual average of 22 ± 6.4 μg/m^3^ in 1981 to 7.9 ± 2.1 μg/m^3^ in 2016 due in no small part to 1970 amendments to the Clean Air Act.^2^ However, ambient air pollution in the United States has stabilized or in some locales increased likely as a direct result of wildfires since 2016.^3^ In the summer of 2023, wildfires across Canada and United States enshrouded much of the population in North America in a miasma of smoke and air pollutants, highlighting a reverse in the trend of improving air quality. Due to climate change, conditions needed to cultivate wildfire are anticipated to become more common seasonally and widespread geospatially.^4^ Thus, we can expect PM_2.5_ will have increasing relevance as a cardiovascular risk factor worldwide in the coming years.

As the authors Deo et al^5^ point out, PM_2.5_ is a well-established risk factor for cardiovascular disease as a whole and atherosclerosis in particular.^6^ The mechanisms for accelerating atherosclerosis are broad and include endothelial dysfunction, inflammation, coagulation and thrombosis, vasoconstriction, and plaque instability with oxidative stress mechanisms functioning as key mediators.^1^ It is on this body of basic and translational research linking ambient air pollution exposure to atherosclerosis and cardiovascular events that epidemiological studies used to evaluate the human toll of exposure demonstrate statistical relationships that are plausibly causal. Landmark epidemiologic studies in human populations suggest an approximate 20% to 30% increase in ischemic heart disease mortality for each 10 μg/m^3^ increase in PM_2.5_ exposure when adjusted for a variety of regional and socioeconomic variables.^7^^,^^8^ Even short PM_2.5_ exposure duration is important with meta-analytic data suggesting that every 10 μg/m^3^ of PM_2.5_ exposure in a *single day* increases the relative risk of myocardial infarction by 2.5% (OR: 1.025 [95% CI: 1.015-1.036]).^9^ This finding has a staggering impact when we consider the PM_2.5_ levels the U.S. population from Chicago to Washington, D.C., was subjected to in June 2023.

Of course, incident myocardial infarction commonly leads to revascularization with either percutaneous interventions or coronary bypass. Importantly, the effects of persistent PM_2.5_ exposure after intervention are not well studied. Recent work established PM_2.5_ as a risk factor for events following percutaneous coronary intervention^10^ which begs the question if there are similar risks after coronary bypass revascularization.

Deo et al^5^ presented a statistically rigorous study analyzing the association between PM_2.5_ and cardiovascular events after coronary artery bypass surgery adding to recent work establishing PM_2.5_ as risk factor for events following percutaneous coronary intervention. This study harnesses the relative strengths of veterans affairs data with large numbers of meticulously adjudicated bypass recipients with medical comorbidities. The findings mirror prior reports in patients without revascularization with a fully adjusted relative risk of 18% increase in cardiovascular events. The demographics of the study are largely representative of bypass recipients nationally, with a notable exception of few included women, who may not share a similar benefit from bypass surgery.^11^ Whether or not improved adherence to smoking cessation recommendations (20%-25% of the study population reported to be smokers) or more aggressive lipid control (low-density lipoprotein levels of 90 to 95 mg dL across included patients) reduces the impact of PM_2.5_ exposure remains unknown and requires further investigation since control of conventional risk factors may diminish the impact of ambient air pollution exposure.

The authors hypothesize that limiting PM_2.5_ exposure to a maximum mean of 8 μg/m^3^ would result in a 1.7% absolute risk reduction in cardiovascular events and further lowering this level to 5 μg/m^3^ eliminates the lion’s share of PM_2.5_ attributable cardiovascular events. This analysis provides an aspirational policy target with the potential for widespread benefits to the population as a whole. For additional perspective, lowering low-density lipoprotein levels to a mean of roughly 30 mg/dL with evolocumab in high-risk patients enrolled in the Fourier trial resulted in a 1.5% absolute risk reduction in the composite endpoint.^12^ Of course, the standard limitations to any non randomized control trial apply here and real-world data for a fair comparison is not yet available but the results of this study are provocative and suggest clinicians should take this risk factor seriously and think beyond standard risk factors in high-risk patients.

Though randomized trial data quantifying the magnitude of risk reduction for interventions aimed at this omnipresent risk factor are lacking, some impactful strategies may be relatively straightforward to deploy. Indeed, a randomized crossover trial showed a high efficiency face mask reduced angina and ST-segment depressions in participants with pre-existing coronary artery disease asked to walk through Beijing on a particularly polluted day (PM_2.5_ 70 μg/m^3^).^13^ Even in healthy adults, the use of a respirator and home air filter resulted in small reductions in systolic blood pressure and improved inflammatory biomarkers.^1^ Though formal recommendations might be premature with outcomes data still lacking, clinicians would not be remiss in suggesting exposure mitigation strategies such as masking or home air filtration for particularly high-risk patients on polluted days.

Cardiovascular risk factor modification must be inclusive of not just pathophysiologic factors like insulin resistance or hyperlipidemia but should also be contextualized in socioeconomic status and environmental exposures. To address the risk that we cannot see, individual risk assessment for cardiovascular disease must be expanded beyond Framingham to include societal, economic, and environmental factors that shape outcomes. Future investigations should trial strategies for high-risk patients, like pollution exposure mitigation, to protect patients from this enduring and pervasive risk factor.

## Funding support and author disclosures

The author has reported that they have no relationships relevant to the contents of this paper to disclose.
